# The complete chloroplast genome of the brown alga *Saccharina* sp. ye-B (Laminariaceae: Phaeophyceae) from Sakhalin, Russia

**DOI:** 10.1080/23802359.2021.1878964

**Published:** 2021-02-19

**Authors:** Yan Li, Xiao Fan, Wei Zhang, Dong Xu, Yitao Wang, Xiaowen Zhang, Naihao Ye

**Affiliations:** a College of Fisheries and Life Science, Shanghai Ocean University, Shanghai, PR China; b Yellow Sea Fisheries Research Institute, Chinese Academy of Fishery Sciences, Qingdao, PR China

**Keywords:** Complete chloroplast genome, Illumina sequencing, *Saccharina* sp. ye-B

## Abstract

The complete chloroplast genome sequence of *Saccharina* sp. ye-B was determined using Illumina sequencing data. The chloroplast genome of *Saccharina* sp. ye-B is 130,587 bp in length, containing 139 protein-coding genes (PCGs), 3 ribosomal RNAs (rRNAs), and 28 transfer RNAs (tRNAs) genes. The phylogenetic reconstruction based on the chloroplast genomes of 11 brown algae resolves *Saccharina* sp. ye-B in a fully supported clade with *S. japonica*. The determination of the chloroplast genome of *Saccharina* sp. ye-B will benefit future algal genetics, evolution, and systematic studies in the Laminariaceae.

*Saccharina* is a marine genus of Phaeophyceae with great economic importance. Due to its commercial use as a raw material in food and industry (Kim et al. 2014), it is regarded as one of the most common seaweeds cultivated in China, Japan, and Korea (Hwang et al. 2017). However, there is a problem of degradation in germplasm resources of kelp due to inbreeding in China. Aiming to improve the genetic variance and economic value of *S. japonica*, *Saccharina* was collected worldwide. Here, we determined the complete chloroplast genome sequence of a wild strain of *Saccharina* collected from Sakhalin, Russia (46°2′16.4′′N, 143°28′29.2′′E). The strain was subsequently named *Saccharina* sp. ye-B based on the phylogenetic analysis with 11 brown algae chloroplast genomes. The phylogeny of the strain would provide a genetic source for further studies on the phylogenetic relationships and molecular studies of the genus *Saccharina*.

DNA was isolated using a modified phenol-chloro procedure (Greco et al. 2014). Illumina sequencing of this DNA yielded 152 million, clean PE 100 reads. Approximately 32% of the total number of reads were mapped to the reference chloroplast genome *S. japonica* (NC018523, 130,584 in length) using Burrows–Wheeler Aligner (BWA) (Li and Durbin 2009). No gaps were found in the mapping result. The consensus sequence was obtained using Geneious version 11.1.4 (http://www.geneious.com). The annotation was performed using BLAST (Altschul et al. 1997), DNA star (Burland 2000), and DOGMA (Wyman et al. 2004) as well as checked manually.

The total length of the chloroplast genome sequence of *Saccharina* sp. ye-B is 130,587 bp. The complete chloroplast genome contains 139 protein-coding genes (PCGs), 3 ribosomal (rRNA) genes (5S rRNA, 16S rRNA, 23S rRNA, and two copy for each), and 28 transfer (tRNA) genes. The genome structure, gene content, and gene order were well conserved compared to other brown algae (Fan et al. 2020). The Nucleotide frequency of the H-strand is as follow: T, 34.5%; A, 34.4%; C, 15.4%; and G, 15.7%. The chloroplast of *Saccharina* sp. ye-B encodes 32,367 amino acids, including the start codons. The rRNAs of the two 5S rRNAs are 110 and 100 bp, respectively, the two 16S rRNAs are both 1480 bp and the two 23S rRNA genes are 2944 and 2946 bp in length, respectively. The complete chloroplast genome of *Saccharina* sp. ye-B was deposited at GenBank (MW038824).

The phylogenetic tree was reconstructed with Bayes and maximum likelihood optimality criteria based on the concatenated protein sequences shared among *Saccharina* sp. ye-B and other 11 brown algae complete chloroplast genomes using IQtree2 (Minh et al. 2020). All nodes were full supported based on the resulting Bayesian posterior probabilities and 1000 Bootstrap replicates. The phylogenetic analysis showed that *Saccharina* sp. ye-B was resolved in a clade with *S. japonica*, in a sister position to *S. lattisima* (Figure 1). These results are consistent with the phylogeny derived from the mitochondrial genome (Guan et al. 2014).

**Figure 1.**
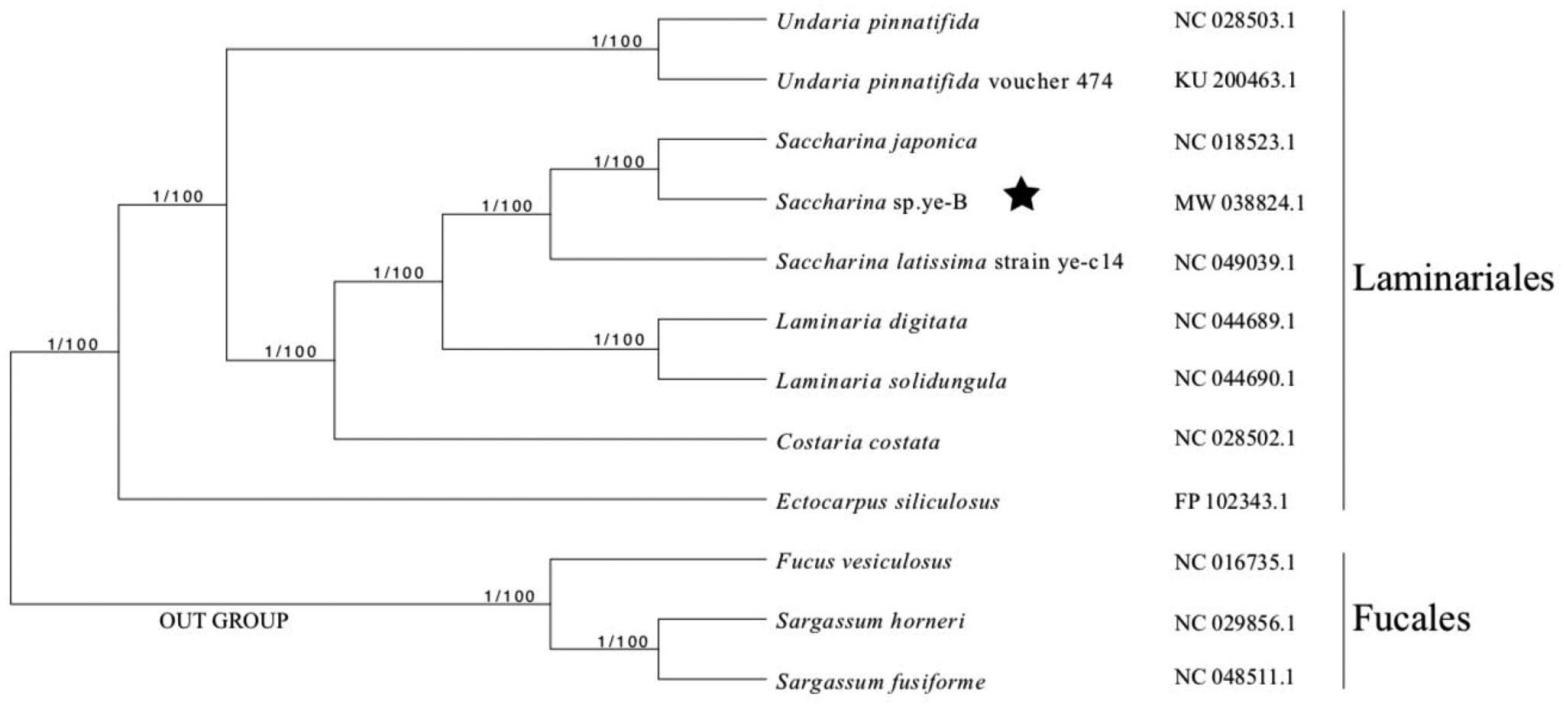
Bayes/maximum likelihood phylogenetic tree of *Saccharina* sp. ye-B with other species in the blown algae based on concatenated protein sequences. Numbers in the nodes are support values of Bayes test and the ML bootstrap from 1000 replicates. *Fucus vesiculosus*, *Sargassum horneri*, and *Sargassum fusiforme* were set as the out group. The pentagram stands for the new sequenced species in our work.

## Data Availability

Chloroplast data supporting this study are openly available in GenBank at nucleotide database, https://www.ncbi.nlm.nih.gov/nuccore/MW038824. Associated BioProject, https://www.ncbi.nlm.nih.gov/bioproject/PRJNA272647, BioSample accession number at https://www.ncbi.nlm.nih.gov/biosample/SAMN03740578 and Sequence Read Archive at https://www.ncbi.nlm.nih.gov/sra/SRX1041447.
